# Factors Associated With Risk Stratification and Overall Survival of Black South African Men With Non‐Metastatic Prostate Cancer

**DOI:** 10.1002/cam4.71628

**Published:** 2026-02-27

**Authors:** Raylton P. Chikwati, Monica Ewomazino Akokuwebe, Olaide O. Ojoniyi, Rebaone Petlele, Shane A. Norris, Audrey Pentz, Maureen Joffe, Sean Doherty, Timothy R. Rebbeck, Wenlong C. Chen

**Affiliations:** ^1^ SAMRC Developmental Pathways for Health Research Unit, Department of Paediatrics University of the Witwatersrand Johannesburg South Africa; ^2^ School of Human Development and Health University of Southampton Southampton UK; ^3^ Strengthening Oncology Services Research Unit, Faculty of Health Sciences University of the Witwatersrand Johannesburg South Africa; ^4^ Network for Oncology Research in Africa (NORA), Global Health Working Group, Martin‐Luther‐University Halle‐Wittenberg Germany; ^5^ Division of Urology, Department of Surgery, Faculty of Health Sciences University of the Witwatersrand Johannesburg South Africa; ^6^ Harvard TH Chan School of Public Health and Dana‐Farber Cancer Institute Boston Massachusetts USA; ^7^ Sydney Brenner Institute for Molecular Bioscience, Faculty of Health Sciences University of the Witwatersrand Johannesburg South Africa; ^8^ National Cancer Registry, A Division of the National Institute for Communicable Diseases, National Health Laboratory Service Johannesburg South Africa

**Keywords:** non‐metastases, overall survival, prostate cancer, South Africa

## Abstract

**Background:**

Emerging evidence indicates significantly poorer overall survival for men with metastatic prostate cancer in resource‐limited settings than in high‐income countries. However, there is less understanding of the overall survival of non‐metastatic disease, which could inform early treatment strategies.

**Objective:**

To prospectively examine factors associated with the National Comprehensive Cancer Network (NCCN) risk stratification and overall survival in 741 Black South African men with non‐metastatic prostate cancer, some of whom also had co‐morbidities (≥ 2 other chronic conditions).

**Methods:**

Baseline data on social and health factors were collected. Follow‐up of participants monitored overall survival over a median of 4.3 (3.5–5.0) years. We used multivariable proportional ordinal regression to examine factors associated with non‐metastatic prostate cancer risk stratification. Kaplan‐Meier, Cox proportional hazards regression, and Pohar‐Perme methods were used to calculate overall survival and assess associations.

**Results:**

Our findings showed a generally favourable prognosis of non‐metastatic prostate cancer with a 5‐year overall survival of 79.0% (75.6–82.6) while the 5‐year age‐standardised net survival was 91.0% (95% CI 86.0–97.0). Overall survival differed significantly by the different NCCN risk groups, emerging early and widening over time, with the lowest survival in the high‐risk groups. Only older age at diagnosis (Hazard Ratio per one‐year increase:1.05 (95% CI: 1.02–1.08)), diabetes (HR: 1.70 (95% CI: 1.08–2.67)), and depression (HR: 1.67 (95% CI: 1.09–2.57)) at study recruitment were associated with poorer overall survival. Furthermore, only older age at diagnosis (HR: 1.04 (95% CI: 1.02–1.07)) was associated with higher non‐metastatic prostate cancer risk.

**Conclusions:**

These findings emphasise the need to address early diagnosis and comorbidities in non‐metastatic prostate cancer, which could improve overall survival.

**Abbreviations**
ADT: androgen‐deprivation therapy
ANOVA: analysis of variance
BMI: body mass index
BP: blood pressure
CHBAH: Chris Hani Baragwanath Academic Hospital
ELISA: enzyme‐linked immunosorbent assay
GHO: WHO Global Health Observatory
HIV: human immunodeficiency virus
HR: hazard ratio
IARC: International Agency for Research on Cancer
ICSS: International Cancer Survival Standard
IQR: interquartile range
LHRH: luteinising hormone‐releasing hormone
LMICs: low and middle‐income countries
MADCaP: Men of African Descent and Prostate Cancer Consortium
MVPA: moderate‐to‐vigorous physical activity
NCCN: National Comprehensive Cancer Network
PHQ‐9: Patient Health Questionnaire‐9
TNM: tumour, nodes, metastasis system
VIFs: variance inflation factors

## 1 Introduction

Prostate cancer is a growing global health problem with an estimated 1.5 million new cases and 397,000 deaths in 2022, posing a significant threat to men's health [1]. However, the global incidence and mortality have varied across different regions [1, 2]. According to the International Agency for Research on Cancer (IARC) 2022 report, high‐income countries have higher incidence compared to low and middle‐income countries (LMICs), while the opposite is true for mortality [1]. In Northern Europe, for example, the age‐standardised incidence was 82.8 cases per 100,000 men, whereas in Southern Africa, 59.9 cases per 100,000 men were reported. Regarding age‐standardised mortality, Northern Europe had 12.4, compared to 29.7 cases per 100,000 men in Southern Africa [1]. These disparities may reflect inequities in the early diagnosis, management and prognosis of prostate cancer.

**FIGURE 1:**
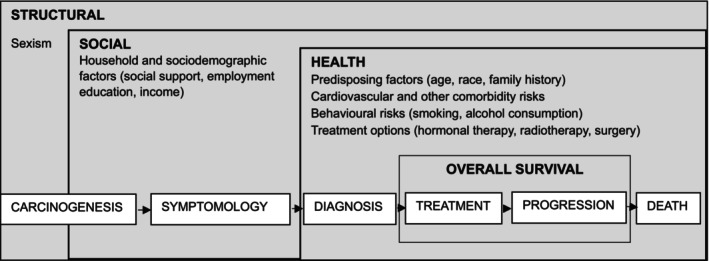
Conceptual framework for translating health services, research and interventions into solutions to address inequities in prostate cancer care and overall survival (adapted from Nyame et al. [3]).

A conceptual framework identifying the main drivers associated with these inequities is necessary to enhance our understanding of the complexities underlying disease risk and overall survival. While common risk factors for prostate cancer, such as older age and family history, are well‐characterised, modifiable factors, including structural, social and other health factors, have now been recognised in a recent conceptual framework (Figure 1) [3]. This framework, leverages the evolution of prostate cancer risk factors and the importance of their combined effect towards incidence and survival [3].

Studies in the US show that African Americans face more health disparity challenges and are more prone to prostate cancer than their White counterparts [4, 5, 6]. While there is little data on the direct comparisons between African Americans and Black South Africans (or other sub‐Saharan African populations), Tindall et al. (2014) showed that South African Black men are at a 2.1‐fold higher risk for advanced prostate cancer at presentation compared to African Americans [7]. Recent findings show that Black South Africans have a higher risk of being diagnosed with aggressive disease, including a greater risk than men of mixed ancestry in South Africa [8].

In South Africa, the 2023 National Cancer Registry (NCR) reported that prostate cancer contributed to about 25.9% of all male cancer [9]. Age‐standardised incidence was about 47.0 per 100,000 men, representing a total of 10,944 new cases in 2022, with marked racial differences (White men: 76.9; Black men: 39.1; men of mixed ancestry: 53.3; Asian men: 27.7 per 100,000), likely underestimations largely driven by health disparity challenges. 12 Evidence from IARC shows an increase in prostate cancer mortality in South African men between 2020 and 2022, from about 22.1 per 100,000 in 2020 to 30.7 per 100,000 in 2022, highlighting a growing national burden [10, 11]. While there are no population‐wide or registry‐based data, the median age of prostate cancer diagnosis from hospital‐based cohorts ranges from 66 to 68 years [12, 13, 14]. Substantial disparities in incidence and aggressiveness of prostate cancer have been reported in South Africa. A 10‐year retrospective study using prostate biopsies showed that Black South African men accounted for the highest proportion (46%) of high‐grade disease, compared to White (36%), Asian (38%) and mixed ancestry (40%) populations [15]. These disparities between incidence and high‐grade disease therefore necessitate a need to identify risk factors that could help inform early detection and guide treatment decisions, particularly in Black South African men.

The National Comprehensive Cancer Network (NCCN) risk stratification is a widely used clinical tool for categorising non‐metastatic prostate cancer into either low, intermediate, or high risk, based on prostate‐specific antigen (PSA) levels, Gleason scores (from biopsy) and tumour staging (using the tumour nodal metastatic system (TNM)) [16]. The TNM system aids in determining the local (T), regional (N) and distant (M) spread of the cancer.

In sub‐Saharan Africa, research on prostate cancer survival is limited, with studies focusing primarily on metastatic disease [17, 18]. Our group has previously described metastatic disease and factors associated with overall survival [13]. Together, these studies only address the challenges faced in treating metastatic disease, including limited access to screening and treatment, which impact survival outcomes. However, there is still a scarcity of survival data on non‐metastatic disease. Furthermore, challenges such as small sample sizes and difficulties with long‐term follow‐ups continue to hinder our understanding of prostate cancer survival data in the region. Recently, the Lancet Commission on prostate cancer called for more region‐specific data, especially in sub‐Saharan Africa, which is currently underrepresented in global prostate cancer studies and yet faces higher rates of aggressive disease [19].

The aim of this study was to identify factors associated with prostate cancer risk stratification and to identify factors associated with 5‐year overall survival among Black South African men.

## 2 Methods

### 2.1 Study Population

This study is part of the Men of African Descent and Prostate Cancer (MADCaP) Consortium [20], which recruited self‐identifying Black African men from outpatient clinics at the Chris Hani Baragwanath Academic Hospital (CHBAH) in Soweto, Johannesburg. The CHBAH predominantly serves patients who are referred from local community‐based primary care clinics in greater Soweto, an urban community in southern Johannesburg. Study recruitment was from November 2016 to July 2020. Follow‐up of participants continued until the earliest of the following: the date of death, the last known date the participant was alive, 5 years after diagnosis, or 7 November 2022, whichever occurred first. Only the final months of recruitment occurred during the COVID‐19 pandemic and lockdown in South Africa (March 2020–June 2022), and approximately 2 years of the 5‐year follow‐up took place within this period.

### 2.2 Participant Recruitment and Inclusion Criteria

Study participants were eligible for recruitment if they self‐identified as Black African, were 30 years of age or older, provided informed and written consent, and had a newly confirmed diagnosis of prostate cancer through histological examination. Participants were required to reside within the catchment area surrounding the CHBAH study centre. Only participants with an incident prostate cancer diagnosis within 6 months prior to study contact were eligible for inclusion. We only included participants with confirmed non‐metastatic disease as defined by the NCCN risk stratification of low, intermediate, high and very high‐risk categories [16]. Participants with metastatic prostate cancer were excluded to minimise heterogeneity in survival outcomes.

### 2.3 Data Collection

At enrolment, face‐to‐face interviews were conducted to collect data on social (household and sociodemographic factors, including social support, employment, education and income) and health factors (age, family history, comorbidity risks and behavioural risks). Family history of prostate cancer was assessed based on self‐reported history in first‐degree male relatives, including the participant's father, brothers and sons. Depression was assessed using the Patient Health Questionnaire‐9 (PHQ‐9) [21]. Derived scores were categorised into absent (0–4), mild (5–9) and moderate to severe (> 10) as defined by Zimmerman [22]. Measurements on height, weight and waist circumference were also taken using standard procedures. Body mass index (BMI) was calculated by dividing weight in kilogrammes by the square of height in metres. Diabetes status was assessed based on self‐report, defined as either taking glucose‐lowering medication or having a prior diagnosis of high blood glucose. Blood pressure (BP) measurements were taken from seated participants, using the average of the last two readings from a total of three. Hypertension was defined as a systolic BP of 140 mmHg or more, or a diastolic BP of 90 mmHg or more, or a previous diagnosis by a health‐care professional [23]. All participants were tested for HIV using the enzyme‐linked immunosorbent assay (ELISA).

Moderate‐to‐vigorous physical activity (MVPA) was estimated from the self‐reported data on weekly activity time. Insufficient physical activity was defined as MVPA < 150 min/week [24]. Weekly alcohol consumption (grams) was estimated by converting self‐reported frequency of beer, wine, liquor and traditional alcohol intake using standard drink volumes, ethanol concentration and density. Total alcohol intake was then calculated by summing grams across beverage types, and heavy alcohol consumption was defined as > 168 g/week.

Prostate cancer diagnoses were confirmed through core biopsies and Gleason scores. PSA levels were routinely measured at diagnosis. Clinical T‐staging was performed using digital rectal examinations following the standard protocol in our resource‐limited public tertiary hospitals. Non‐metastatic low‐risk disease was characterised by a PSA level below 10 ng/mL, Epstein‐Gleason Grade Group 1 (Gleason score 3 + 3) and a clinical stage of T1‐T2a. Intermediate high‐risk disease was defined as a clinical stage T2b or T2c, and/or Epstein Grade Group of 2 or 3 (Gleason score 3 + 4 or 4 + 3), and/or a PSA level between 10 and 20 ng/mL. High‐risk disease included a clinical stage T3a, a Gleason score of 8–10 regardless of PSA levels, or a PSA level exceeding 20 ng/mL. Very high‐risk disease was defined as a clinical stage T3b, T3c or T4. The high‐ and very‐high‐risk categories were merged into a single high‐risk group. Participants received androgen‐deprivation therapy (ADT) with luteinising hormone‐releasing hormone (LHRH) agonists and/or oral non‐steroidal first‐generation antiandrogens.

Vital status was confirmed by telephone after every 3 months. If the participant, next of kin, or other person named as close contacts could not be reached for two consecutive follow‐up calls, we searched VerifyID (www.verifyid.co.za), a publicly available administrative database, to determine the participant's vital status. Participants were censored at the last date when they were known to be alive if no additional information about vital status could be obtained.

### 2.4 Study Outcomes

Our primary outcome was overall survival. Secondary outcomes included the factors associated with non‐metastatic prostate cancer risk stratification and overall survival.

### 2.5 Statistical Analyses

Baseline characteristics on social and health risks were summarised according to the three non‐metastatic prostate cancer risk categories, using appropriate descriptive statistics. The Shapiro–Wilk test was used to assess the normality of continuous variables, and these were expressed as mean ± standard deviation (if normally distributed) and median (interquartile range) if not normally distributed. Categorical variables were presented as proportions (sample size, percentage). One‐way Analysis of Variance (ANOVA) was used to compare the means of continuous variables across the three risk categories if the data were normally distributed. The Pearson's chi‐square test was used to assess the associations across categorical data. We applied the Bonferroni correction by dividing the *p*‐value: 0.05 threshold for significance by the number of hypothesis tests conducted in the descriptive analyses.

Proportional ordinal regression was used to examine the association between baseline socio‐demographic, co‐morbidity, behavioural and treatment data across the three non‐metastatic prostate cancer risk categories. All variables were included together in a single multivariable proportional ordinal regression model. Variance inflation factors (VIFs) in the adjusted model were all below 2, indicating no significant multicollinearity. Survival analyses were conducted on a time‐since‐diagnosis scale, with the at‐risk period starting on the date of histologically confirmed diagnosis of prostate cancer until the end of follow‐up. Kaplan–Meier survival curves stratified by the three prostate cancer risk categories were used to assess overall survival in the full cohort. Survival comparisons were performed using the log‐rank test. Cox proportional hazards regression was used to examine the association between baseline socio‐demographic, co‐morbidity, behavioural and treatment factors on overall survival. Considering we used overall survival as the outcome variable; stratified Cox proportional hazards regression was performed by using the median age (66 years) and creating two groups: participants aged < 66 years and those aged ≥ 66 years. To evaluate whether associations differed by age, we fitted a combined model by including centred age (difference between age at diagnosis and median age) and interaction terms between median age groups and significant factors. We did a complete case series analyses under the assumption that data were missing at random. Variables with high missingness, that is, family history of prostate cancer and household income were excluded from regression analyses. The extent of missingness in all independent variables included in the regression models was evaluated and found to be minimal; therefore, multiple imputation was unlikely to meaningfully influence the estimated effects. To adjust for survival due to causes of death other than prostate cancer, we estimated net and age‐standardised net survival by accounting for background age‐specific national mortality. Net and age‐standardised net survival were estimated using the Pohar‐Perme method using the *rs.surv()* function from the *relsurv* R package, with expected mortality derived from national life tables. Age standardisation was performed using the International Cancer Survival Standard (ICSS) weights [25]. We obtained age‐specific death rates for South African men from the 2020 WHO Global Health Observatory (GHO) and Life Tables. Age‐specific death rates by age group were smoothed into continuous age‐specific death rates using a flexible Poisson model, as described by Rachet et al. [26].

The level of statistical significance was set at a two‐tailed *p <* 0.05. All statistical analyses and visualisations were performed using Stata 18.0 (StataCorp LLC, College Station, TX, USA) and RStudio 4.0 (RStudio Team, PBC, Boston, MA, USA).

## 3 Results

### 3.1 Social and Health Factors at Study Recruitment

A total of 741 men with non‐metastatic prostate cancer were recruited between November 2016 and July 2020. Only 17 (2.3%) of men were recruited during the COVID‐19 pandemic. A total of 189 (25.5%) of these men had Epstein Grade Group 4 and 5 tumours; 23 (3.1%) had T3 staging, and 303 (40.9%) had levels of PSA > 20 ng/mL at diagnosis. For risk stratification, we excluded 6 (0.8%) participants who had missing data on PSA (*n* = 5), T staging (*n* = 1) and grading (*n* = 1); one participant had missing data on two variables (PSA and T staging). In total, 735 participants were included in the analyses, with 58 (7.8%) as low risk, 305 (41.2%) intermediate risk and 372 (50.8%) high risk non‐metastatic prostate cancer cases (Table 1).

**TABLE 1:** Comparison of risk categories of non‐metastatic prostate cancer in study cohort according to social and health factors (*n* = 735).

Exposure domain		Risk			*p*
*N* (%)		Low risk	Intermediate	High/Very high	
Mean ± SD					
Number of men enrolled		58 (7.9)	305 (41.3)	372 (50.8)	—
Age risk					
Age at diagnosis		62.8 ± 8.2	65.2 ± 8.1	66.7 ± 7.6	0.564
Epstein grade group	1	58 (100.0)	29 (9.6)	15 (4.0)	—
	2 or 3	NA	274 (90.4)	170 (45.7)	—
	4 or 5	NA	NA	187 (50.3)	—
T stage	T1 (1a‐1c)	54 (93.1)	215 (71.2)	187 (50.3)	—
	T2 (2a‐2c)	4 (6.9)	87 (28.8)	162 (43.5)	—
	T3 (3a‐3c)	NA	NA	23 (6.2)	—
PSA (ng/mL)	≤ 10	58 (100.0)	146 (48.2)	34 (9.1)	—
	10–20	NA	157 (51.8)	36 (9.7)	—
	> 20	NA	NA	302 (81.2)	—
Household and sociodemographic vulnerability risk					
Minimal social support (single vs. cohabiting)					
Single (including divorced and widowed)		15 (25.9)	84 (27.5)	113 (30.4)	0.628
Employment status (unemployed vs. employed)					
Unemployed (including retired and students)		46 (79.3)	249 (81.6)	316 (85.0)	0.363
Education level (≤ primary vs. secondary or higher)					
Primary school or less (R0/G7 or informal)		36 (62.1)	187 (61.3)	233 (62.6)	0.940
Household income per month (low to middle wealth score vs. high)^a^					
Low to mid wealth index score (0–3)		53 (91.4)	283 (92.8)	344 (92.5)	0.614
Missing		5 (8.6)	22 (7.2)	27 (7.3)	
Family history (present vs. absent)					
Yes		12 (20.7)	15 (4.7)	24 (7.5)	—
Missing		32 (55.2)	168 (55.1)	213 (57.3)	
CVD and other comorbidity risks					
Waist circumference, cm		95.8 ± 12.4	95.5 ± 12.4	95.3 ± 12.0	0.953
BMI (kg/m^2^)		27.3 ± 5.2	27.1 ± 5.3	27.1 ± 5.5	0.952
Morbid obesity		3 (5.2)	21 (6.9)	31 (8.3)	0.613
Diabetes		9 (15.5)	56 (18.4)	51 (13.7)	0.245
Hypertension		41 (70.7)	212 (69.7)	252 (67.7)	0.839
Heart disease		3 (3.5)	7 (2.3)	4 (1.1)	—
CVD risk comorbidity burden (morbid obesity, diabetes, hypertension)					
≥ 1 comorbidity		42 (72.4)	221 (72.7)	261 (70.2)	0.756
Living with HIV (no vs. yes)					
Yes		9 (15.5)	29 (9.5)	51 (13.7)	0.181
Missing		0 (0.0)	2 (0.7)	2 (0.5)	
Depression					
0–4: No psychological distress		51 (87.9)	260 (85.3)	317 (43.1)	0.976
5–8: Mild distress		4 (6.9)	31 (10.2)	37 (10.0)	
9–12: Moderate distress		2 (3.5)	11 (3.6)	12 (3.2)	
13+: Severe distress		1 (1.7)	3 (1.0)	6 (1.6)	
Behavioural risks					
Physical inactivity (< 150 min/week)		1 (7.1)	10 (3.3)	16 (4.3)	—
Missing		44 (75.9)	253 (83.0)	326 (87.4)	
Tobacco smoking					
Currently yes		16 (44.8)	90 (29.5)	107 (28.8)	0.937
Yes (in the past)		21 (36.2)	119 (39.0)	138 (37.1)	
Never		21 (36.2)	96 (31.5)	127 (34.1)	
Alcohol consumption					
Currently yes		26 (44.8)	146 (47.9)	144 (38.7)	0.057
Yes, in the past		13 (22.4)	93 (30.5)	124 (33.3)	
No, never drank alcohol		19 (32.8)	66 (21.6)	104 (28.0)	
Alcohol consumption per week (heavy vs. none to moderate)					
Heavy alcohol consumption (> 168 g/week)		36 (10.0)	135 (37.9)	186 (51.8)	0.039
Treatment					
Hormonal treatment (no vs. yes)					
Yes		8 (13.8)	156 (51.2)	356 (95.7)	< 0.001
Missing		0 (0.0)	3 (1.0)	0 (0.0)	

*Note:* NCCN risk stratification: low risk – T stage T1–T2a, Epstein Grade Group 1, PSA < 10 ng/mL. intermediate risk: T2b–T2c, Epstein Grade Group 2 or 3, PSA 10–20 ng/mL and high/very high risk: T3–T4, Epstein Grade Group 4 or 5, PSA > 20 ng/mL. Epstein Grade Group 1 (Gleason Score ≤ 6; ≤ 3 + 3), Grade Group 2 (Gleason Score 7; 3 + 4), Grade Group 3 (Gleason Score 7; 4 + 3), Grade Group 4 (Gleason Score 8; 4 + 4, 3 + 5, 5 + 3), Grade Group 5 (Gleason Score ≥ 9; 4 + 5, 5 + 4, 5 + 5), T stage (tumour stage—The numbers and letters describe how far the primary tumour has spread in and around the prostate); T1: tumour not clinically detectable, T2: tumour confined within the prostate, T3: tumour has extended outside the prostate.

Abbreviations: NA, not applicable; PSA, prostate specific antigen.

^a^ Household income score categories: low to middle wealth 0 = up to 1850 per month, 1 = 1851–10,000 per month, 2 = 10,001–30,000 per month, high wealth score > 30,000 per month; currency in South African Rands, morbid obesity = BMI ≥ 35 kg/m^2^, heavy alcohol consumption—consumption of beer + wine + liquor + other per week, hormonal treatment—androgen deprivation therapy. Variables were excluded from the table if missingness was < 0.5% across all risk categories and variables with ≥ 0.5% missingness in any group were retained for transparency. Level of significance after Bonferroni correction: *p* < 0.003.

The age at diagnosis for the cohort was 65.8 ± 7.9 years, with ages ranging from 37 to 87 years. Participants had a mean BMI of 27.1 ± 5.4 kg/m^2^. The highest prevalence of comorbidities included hypertension: 68.6% (95% CI 65.2–71.9), diabetes: 16.0% (95% CI 13.5–18.8), HIV: 12.2% (95% CI 10.0–14.8). When comparing the three risk categories, only hormonal treatment (androgen deprivation therapy) significantly differed between the groups after Bonferroni correction (*p* < 0.003) (Table 1). Men with high‐risk non‐metastatic prostate cancer [356 (95.7%)] received more hormonal therapy than those with intermediate [156 (51.2%)] and low risk [8 (13.8%)]; *p* < 0.001 (Table 1). Furthermore, men with high‐risk disease stratification had markedly elevated PSA levels at diagnosis compared to intermediate and low‐risk groups (median 36.6 vs. 10.5 vs. 6.8 ng/mL; *p* < 0.001). There were no differences between the risk groups in the other socio‐demographic, CVD and other comorbidity risk data, including BMI, waist circumference, HIV status, depression and behavioural risks (alcohol consumption and smoking) (Table 1). Family history and physical activity were excluded from these analyses because of a high level of missing data (Table 1).

### 3.2 Factors Associated With Risk Stratification

**FIGURE 2:**
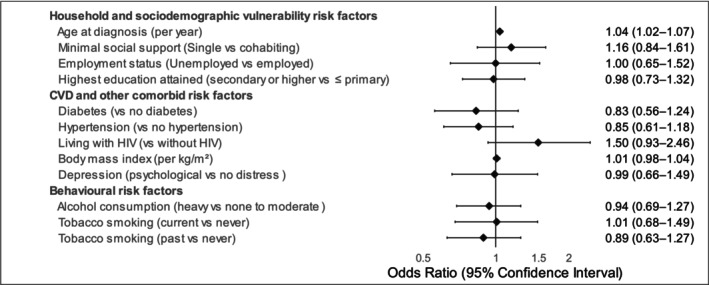
Multivariable proportional ordinal regression on non‐metastatic prostate cancer risk stratification (low risk = 0, intermediate risk = 1, high‐risk = 2) and associated risk factors. All variables were included in the single multivariable model. Data are presented as odds ratios (OR) with corresponding 95% confidence intervals (CIs), depicted by symbols and bars, respectively. The vertical solid line passing through 1 demarcates the null effect, with lower risk represented on the left side and higher risk on the right side of the line.

As shown in Figure 2 and Table S1, the multivariable proportional ordinal regression model showed that age at diagnosis was associated with a 4% higher risk of non‐metastatic prostate cancer per 1 year increase in age at diagnosis in the odds of intermediate/high versus low, and high versus low/intermediate risk. No other socio‐demographic, cardiovascular, or comorbidity factors were associated with non‐metastatic prostate cancer risk classification (Figure 2 and Table S1).

### 3.3 Survival

**FIGURE 3:**
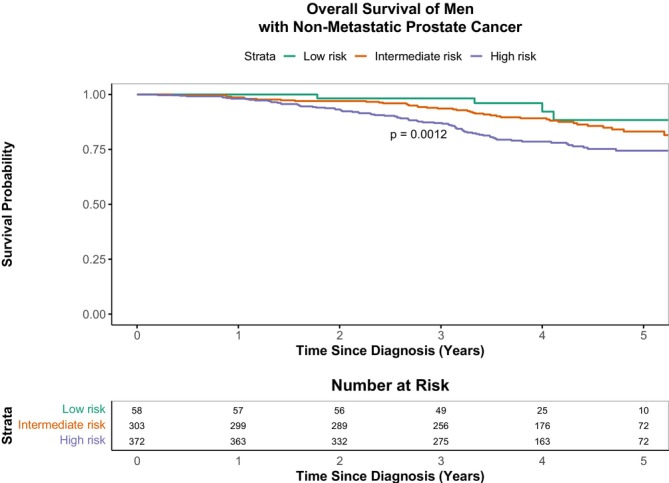
Kaplan–Meier estimates of overall survival according to National Comprehensive Cancer Network (NCCN) risk stratification of non‐metastatic prostate cancer (low, intermediate, and high risk). Differences in overall survival were assessed using the log‐rank test. Level of significance was set at *p <* 0.05. Strata show the sample size of men in the study at each follow up year, and according to the NCCN risk stratification.

A total of 738 men had follow‐up data (irrespective of non‐metastatic prostate cancer risk stratification). From these, 123 (16.7%) died, 151 (20.5%) were alive at administrative censoring, and 461 (62.4%) were censored early (Table 2). A large proportion of participants (461 (62.4%)) were administratively censored before 5 years because the database was closed on 7 November 2022. The median age (IQR) of deceased men during the 5‐year follow‐up period in years was 71 (59–87) years. The median follow‐up was 4.3 (3.5–5.0) years. Overall, mortality peaked in years 3–4, when 36 men (4.9%) died, reflecting the generally high survival of participants with non‐metastatic prostate cancer and the limited number of participants for which follow‐up beyond this time was available (Table 2, Figure 3 and Figure S1). The 5‐year overall survival was 79.0% (75.6–82.6) (Table 2). Net 5‐year survival estimates were slightly higher than 5‐year overall survival estimates by approximately 4% (absolute differences), and age‐standardised net survival estimates were even higher: 91.0% (86.0–97.0) (Table 2). A total of 733 participants had data on both survival and non‐metastatic prostate cancer risk classification. Figure 3 shows significant differences in overall survival between the three risk stratification groups (log‐rank *p =* 0.001), which emerged at 1 year and widened over time. Furthermore, in sensitivity analyses, the 5‐year overall survival was lowest in the high‐risk group [74.4% (69.4–79.9)], low in the intermediate‐risk group [83.1% (78.1–88.5)], and highest in the low‐risk group [88.4% (77.0–100.0)] (Figure 3).

**TABLE 2:** Follow‐up, death, and survival estimates in the cohort.

	Total
Number of men followed up *N* (%)	738 (99.6)
Mean (SD) age at diagnosis in years	65.8 ± 7.9
Median time since diagnosis IQR, years^a^	4.0 (3.1–4.9)
Median time of follow up IQR, years	4.3 (3.5–5.0)
Status at end of follow‐up^b^	
Died	123 (16.7)
Administrative censoring at 5 years	151 (20.5)
Administrative censoring before 5 years	461 (62.4)
Early censoring (lost to follow up)	3 (0.4)
Number of deaths during time since diagnosis (years)	
0 to < 1	12 (1.6)
1 to < 2	24 (3.2)
2 to < 3	33 (4.5)
3 to < 4	36 (4.9)
4 to ≤ 5	18 (2.4)
Median age (IQR) of deceased men during the 5‐year follow‐up period in years	71 (59–87)
1‐year survival	
Overall survival	98.4 (97.5–99.3)
Net survival^c^	99.0 (98.0–100.0)
Age‐standardised net survival	99.0 (98.0–100.0)
3‐year survival	
Overall survival	90.3 (88.2–92.5)
Net survival^c^	93.0 (91.0–95.0)
Age‐standardised net survival	96.0 (93.0–99.0)
5‐year survival	
Overall survival	79.0 (75.6–82.6)
Net survival^c^	83.0 (79.0–87.0)
Age‐standardised net survival	91.0 (86.0–97.0)

*Note:* Data are *n* (%) or percentage surviving (95% CI) unless otherwise indicated.

^a^ Regardless of vital status.

^b^ End of follow‐up to earliest of 5 years after diagnosis or November 7, 2022, whichever came first.

^c^ Net survival accounts for background age‐specific national mortality in men. Age‐standardised net survival accounts for age group weights based on the International Cancer Survival Standardisation (ICSS) method.

### 3.4 Factors Associated With Overall Survival

**FIGURE 4:**
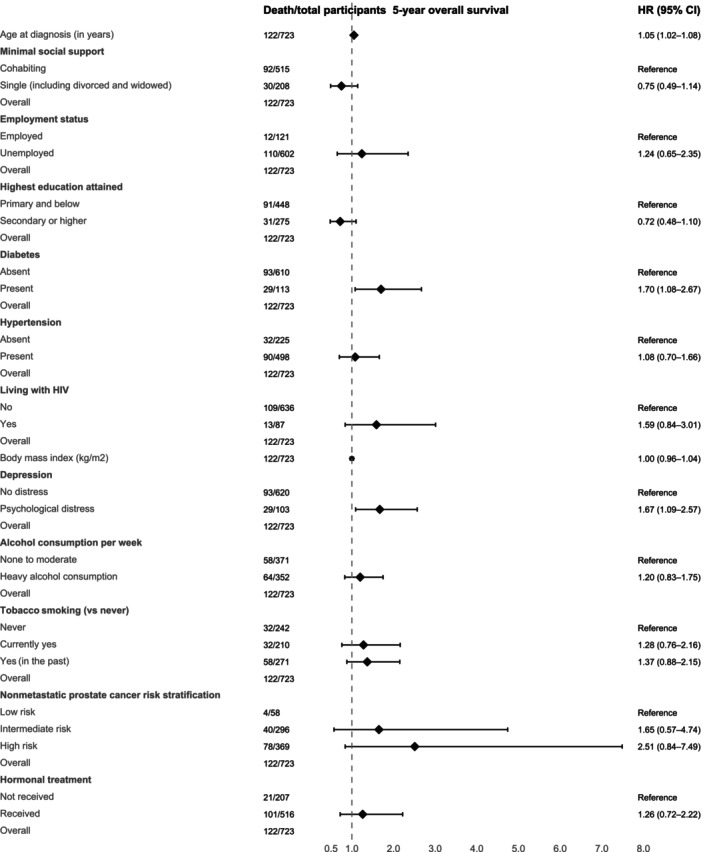
Multivariable Cox proportional hazards regression on overall survival and associated risk factors. All variables were included in the single multivariable model. Data are presented as hazards ratios (HR) with corresponding 95% confidence intervals (CIs), depicted by symbols and bars, respectively. The vertical solid line passing through 1 demarcates the null effect, with HR > 1 indicating higher hazard (i.e., worse overall survival).

In the unadjusted Cox proportional hazards model for overall survival, age at diagnosis (hazard ratio (HR) per 1‐year increase, 1.06, 95% CI 1.03–1.08), high‐risk non‐metastatic prostate cancer (HR = 3.35, 95% CI 1.23–9.16), unemployment (HR = 1.92, 95% CI 1.06–3.48), diabetes (HR = 1.77, 95% CI 1.17–2.69), depression (HR = 2.01, 95% CI 1.33–3.05), past smoking (HR = 1.57, 95% CI 1.02–2.42) and hormonal treatment (HR = 1.99, 95% CI 1.24–3.19) were associated with lower survival. Secondary or higher education (HR = 0.64, 95% CI 0.42–0.96) was associated with higher survival (Table S2). In the multivariable analyses, only age at diagnosis (HR = 1.05, 95% CI 1.02–1.08), diabetes (HR = 1.70, 95% CI 1.08–2.67) and depression (HR = 1.67, 95% CI 1.09–2.57) remained associated with lower survival (Figure 4). No other socio‐demographic, cardiovascular, or comorbidity risk factors were significantly associated with survival (Figure 4 and Table S2).

In the age stratified multivariable analyses, only diabetes (HR = 6.49, 95% 2.83–14.86), depression (HR = 1.67, 95% 1.09–2.57 and hormonal treatment (HR = 6.61, 95% 2.01–21.70) were associated with lower survival among participants aged 60 (57–63) years (Table S3). BMI was associated with higher survival (HR = 0.91, 95%, 0.85–0.98) (Table S3). In older participants aged 71 (68–75) years, only age at diagnosis (HR = 1.06, 95% 1.01–1.11)) and depression (HR = 1.84, 95% 1.09–3.11) were associated with lower survival whereas secondary or higher education (HR = 0.56, 95% 0.32–0.98) was associated with higher survival (Table S4). In a combined model including centred age and interaction terms between median age groups and significant factors, the effects of diabetes*age (interaction HR = 0.91, 95% 0.85–0.97) and hormonal treatment*age (interaction HR = 0.92, 95% 0.87–0.98) on survival decreased with increasing age at diagnosis (Table S5). The main effect of diabetes on survival at median age 66 was high (HR = 2.09, 95% 1.30–3.36). The effects of education*age (interaction HR = 1.00, 95% 0.94–1.05) and BMI*age (interaction HR = 1.00 (0.99–1.01)) were non‐significant, while the main effect of depression on survival remained significant (HR = 1.00 (0.94–1.05)) (Table S5).

## 4 Discussion

Our study highlights a generally favourable prognosis of non‐metastatic prostate cancer with a 5‐year overall survival of 79.0% (75.6–82.6) and a 5‐year age‐standardised net survival of 91.0% (95% CI 86.0–97.0). Survival differed significantly according to NCCN risk stratification, emerging early and widening over time, with the highest mortality in the high‐risk group. Older age at diagnosis, diabetes and depression at study recruitment were associated with higher mortality. Furthermore, only older age at diagnosis was associated with higher non‐metastatic prostate cancer risk stratification.

Our results on the gradient of higher PSA being linked to more advanced disease are consistent with clinical expectation. Cohorts from high‐income countries seldom report risk‐stratified PSA data at diagnosis; however, in one large study on US men managed with active surveillance, median PSA values were 5.35 ng/mL in the low‐risk group, 8.03 ng/mL in the favourable intermediate‐risk group, and 8.63 ng/mL in the unfavourable intermediate‐risk group [27]. In the US Surveillance, Epidemiology, and End Results (SEER) database, about 60% of non‐metastatic cases had PSA < 10 ng/mL, Gleason score ≤ 7, and T‐stage T1–T2b compared to our study which had a much lower proportion of individuals below these thresholds [28]. Comparable high‐risk PSA data are rarely published, underscoring the significance of our findings. In addition, many cohorts from high‐income countries benefit from routine PSA screening, early detection, and access to advanced diagnostics and treatment, which differs markedly from many African contexts including ours.

The present study shares both similarities and distinct differences with other published studies from sub‐Saharan Africa and other LMICs. A previous study consisting of 195 men from 10 sub‐Saharan African countries showed lower 5‐year overall survival in non‐metastatic prostate cancer, where participants with Stage I + II disease (low risk stratification) had overall survival of 64.0% (53.1–77.0) whereas those with Stage III (high risk stratification) had 34.1% (25.3–45.9) [17]. Although the magnitude of difference was different, a similar pattern was observed in a study consisting of 127 men from Sudan, where the 5‐year overall survival for those with stages I and II was 88%, and 57% for stage III [29]. In a meta‐analysis of three studies from India, Indonesia and Brazil consisting of 289 men with non‐metastatic prostate cancer, the 5‐year overall survival after androgen deprivation therapy and radiation therapy was 87% (84.0–94.0) [30]. In a separate analysis, combining 248 patients who had received androgen deprivation therapy, brachytherapy and external beam radiation therapy, the 5‐year overall survival was even higher, at 96% (93.0–98.0) [30]. Furthermore, a systematic review that focused on studies from sub‐Saharan Africa indicated that men diagnosed with non‐metastatic prostate cancer survive longer than those with metastatic disease [31].

A combination of factors may be associated with the observed survival differences between the previously mentioned studies. The differences may reflect variance in staging and treatment of the disease between countries [32, 33]. Furthermore, differences in sample sizes as a result of missing data and inadequate follow‐up may also underestimate survival. The small sample sizes of non‐metastatic cases in most studies from LMICs could suggest a general late presentation for prostate cancer diagnosis. Supporting evidence from a systematic review including 13 studies from sub‐Saharan Africa showed that 6.4%–43.0% of men present for a diagnosis of non‐metastatic prostate cancer [34]. This is in contrast to high‐income countries such as the US, where approximately 70% of men present with non‐metastatic disease [35]. Screening is associated with early detection and therefore is important for identifying low grade tumours in prostate cancer.

The high 5‐year overall survival observed in our study aligns with findings reported in other high‐income countries, such as Norway and the US. From Norway, analyses from nearly 3500 men from the National Cancer Registry with non‐metastatic prostate cancer reported an overall survival of 89.8% (95% CI 88.8–90.8) [36]. When stratified by treatment received, overall survival was lower among patients who did not receive local treatment, at 74.4% (95% CI 72.0–76.6), while those who received radiotherapy with or without adjuvant hormonal therapy were higher (92.6% (95% CI 91.0–93.9)) and even higher in those who received surgery 95.9% (94.3–97.0). In our study, the overall survival of 79.0% (75.6–82.6) was closer to those in the no local treatment group of the Norwegian study. Findings from the SEER Program including over 150,000 patients with high risk non‐metastatic prostate cancer showed an overall 5‐year overall survival of 85.5 (85.3–85.7) [37]. The differences in the magnitude may be related to treatments received between studies. In our study, no participants received surgery, and we could not confirm if participants received brachytherapy or external beam radiation therapy. Our findings of high survival are consistent with our expectation that non‐metastatic prostate cancer is typically indolent, regardless of screening practices. This is interesting because the high survival in our study, an unscreened population was similar to studies from high‐income settings where screening is routine. This underscores the significance of the biology of prostate cancer regardless of screening practices. Consequently, in lower‐income settings where the screening uptake is typically low, aggressive treatment approaches including radiotherapy and surgery may not be warranted. These findings may therefore help strengthen intervention strategies towards more surveillance in low‐risk non‐metastatic prostate cancer and increasing aggressive approaches towards metastatic disease.

The finding that older age at diagnosis was associated with a poorer prognosis of prostate cancer is consistent with global studies. One large study of 10,901 men in the UK with a follow‐up of 14 years showed that older age was associated with a poorer prognosis [38]. This is reflective of the slow, progressive nature of the disease, which makes it more common among older men. The association between age at diagnosis and overall survival could also be indicative of delays in screening because of the location of the prostate, making it difficult for men to disclose their symptoms much earlier. Furthermore, the discomfort of digital rectal examination may discourage elderly men from seeking timely treatment. In the present study, we also observed that there were delays from the time patients received a referral to our study centre, where diagnosis was confirmed.

The present study showed that having diabetes and non‐metastatic prostate cancer at recruitment was associated with a lower 5‐year overall survival. The finding that the association was attenuated between diabetes and survival among older participants warrants further investigations. This finding may reflect limited statistical power due to the relatively small number of diabetic participants in subgroup analyses, despite the prevalence estimates being similar (older: 62 [16.4%]; younger: 53 [15.1%]). Similarly, the finding that hormonal treatment was associated with survival only among the young participants may reflect limited statistical power as suggested by the wide confidence intervals (HR = 6.61, 95% CI 2.01–21.70). Our findings from the total sample and younger participants stratification align with previous meta‐analyses that have shown that pre‐existing diabetes is associated with lower overall survival [39, 40]. It is possible that there is an aggressive interaction between diabetes and prostate cancer, which is associated with increased risk of cardiovascular outcomes and tumour progression. Similarly, our findings indicate that the combined burden of depression and prostate cancer is associated with reduced 5‐year overall survival, also suggesting an adverse interaction between the two conditions. This suggests that personalised treatment for prostate cancer may be warranted for patients with diabetes and/or depression. Moreover, optimising the management of diabetes and depression may be critical to improving overall survival of men diagnosed with prostate cancer.

Although not assessed in this study, we recognise that underlying genetic variation may influence overall survival. Our group has previously published on the contribution of common germline variant risk to prostate cancer in the incidence of variants in Africa. In addition, the HEROIC PCaPH Africa1K has identified rare pathogenic variants in African ancestry, many of which were not captured in standard panels based on non‐African populations [33, 41]. Together, these findings highlight that biological susceptibility to prostate cancer is not uniform and that African populations may harbour distinct genetic architectures that influence both incidence and disease aggressiveness. While these data underscore the importance of ancestry‐specific biological risk factors, we were unable to assess the contribution of biological versus environmental factors on observed survival differences. However, through the same MADCaP Network Consortium, studies are ongoing to uncover unique race specific molecular profiles and correlations to survival outcomes.

Our study had several strengths and limitations. To our knowledge, we had the largest sample size of men with non‐metastatic prostate cancer from South Africa, with a follow‐up duration of 5 years. We collected comprehensive data, including social and health data. We did, however, have some limitations. We had a small number of study participants in the low‐risk prostate cancer group. This was anticipated, as it reflects on the high rates of late presentation and missed opportunities for early diagnosis. However, in a sub‐analysis, we combined the low‐ and intermediate‐risk groups to increase statistical power, and the associations were similar to those we have already presented. The cross‐sectional measurement of psychological distress at enrolment limited our ability to definitively determine whether the PHQ‐9 measure reflected pre‐existing depression or psychological distress triggered by a cancer diagnosis. However, given the nature of how the participants progressed through the healthcare system (i.e., from the primary to the tertiary healthcare facilities where the PHQ‐9 was administered), we hypothesise that the psychological status and responses measured at this point may have been associated with the cancer diagnosis. Future research using longitudinal assessments and broader psychological profiling including mental health history, duration of symptoms and use of antidepressant treatment would be valuable in disentangling these associations. A substantial proportion of participants were administratively censored before accruing 5 years of follow‐up due to closure of the database. Many men were diagnosed late in the enrolment period and had not yet accrued 5 years of potential follow‐up by the data freeze. This pattern reflects rolling enrolment and a limited observation window rather than true loss to follow‐up. Only 0.4% of participants were lost, which shows strong retention and reliable mortality capture. Although this constrained our ability to estimate long‐term survival with precision, administrative censoring is non‐informative and is appropriately handled by Kaplan–Meier and Cox methods; therefore, this limitation is unlikely to bias the overall survival estimates. The overall survival was high (98.4% at 1‐year, 90.3% at 3 years and 79.0% at 5‐years). In a resource limited setting such as ours, disease specific mortality is very difficult to collect robustly. We lacked cause‐specific survival data, and therefore the overall survival may be confounded by non‐cancer mortality. For example, the last months of recruitment occurred during the COVID‐19 pandemic, and around 2 years of follow‐up fell within this period. Therefore, even though the period of overlap with recruitment was small, pandemic‐related mortality may have confounded overall survival. As a result, we were only able to evaluate associations with overall survival but not prostate cancer specific deaths. Although we collected vital status data through follow‐ups and searched VerifyID, a publicly available administrative database, we could retrieve information on living or deceased status and only a few data on cause of death. Consequently, we used overall survival as the outcome, which reflects the combined burden of cancer‐related and unrelated mortality, potentially limiting attribution to prostate cancer alone. To mitigate this limitation, we modelled overall survival using social and health data. We also conducted stratified analyses by age groups to identify factors beyond those associated with ageing. A key limitation was reduced statistical power due to smaller sample sizes within these subgroups. We recognise potential data quality variability due to missing or incomplete values for key variables (including PSA, Gleason score, T‐stage and follow‐up). However, missing data were relatively low to impact effect estimates. We acknowledge that self‐report for a diabetes diagnosis is less accurate than biochemical confirmation as it introduces potential misclassification and under‐detection. We lacked genomic or molecular profiling on participants, which could have uncovered race‐specific tumour biology. Furthermore, our study participants were from local community‐based primary care clinics in greater Soweto, and therefore, the findings may not be nationally representative. Future studies should include diverse regions to better capture national representation, including urban–rural differences. Despite these limitations, our study makes a valuable contribution to the literature as, to the best of our knowledge, it is the largest study to date on risk stratification and survival analyses in non‐metastatic prostate cancer in a sub‐Saharan African population.

In conclusion, our study underscores the importance of early diagnosis of prostate cancer and the need for integrated care in the management of individuals having comorbidities, particularly diabetes and depression. Such targeted interventions may improve overall survival. We have therefore generated valuable data on the overall survival of non‐metastatic prostate cancer from a tertiary hospital in urban South Africa, and this information may be useful for policy formulation towards integrated management of chronic diseases and improving overall survival.

## Author Contributions

**Raylton P. Chikwati:** conceptualisation; methodology; data curation; investigation; validation; formal analysis; data visualisation; writing – original draft; writing – review and editing. **Monica Ewomazino Akokuwebe:** writing – review and editing; validation. **Olaide O. Ojoniyi:** writing – review and editing; validation. **Rebaone Petlele:** writing – review and editing; validation. **Shane A. Norris:** supervision; conceptualisation; methodology; validation; writing – review and editing. **Audrey Pentz:** data curation; writing – review and editing; project administration. **Sean Doherty:** data curation; writing – review and editing; project administration. **Timothy R. Rebbeck:** funding acquisition; review and editing. **Maureen Joffe:** funding acquisition; supervision; methodology; writing – review and editing; project administration. **Wenlong C. Chen:** supervision; data curation; methodology; writing – review and editing; project administration. All authors reviewed and approved the manuscript before submission for publication.

## Funding

This study was funded by an NCI grant U01CA184374 entitled Genetics of Prostate Cancer in Africa. Furthermore, the study was also funded/supported by the Department of Science and Technology and Innovation‐ National Research Foundation (DSTI‐NRF) Centre of Excellence in Human Development at the University of the Witwatersrand, Johannesburg. The project on which this publication is based was in part funded by the German Federal Ministry of Research, Technology, and Space 01KA2220B. This research was funded in part by the Science for Africa Foundation to the Developing Excellence in Leadership, Training and Science in Africa (DELTAS Africa) program [Del‐22‐008] with support from Wellcome Trust and the UK Foreign, Commonwealth & Development Office and is part of the EDCPT2 programme supported by the European Union. The opinions expressed and the conclusions drawn are those of the authors and are not to be attributed to the funders.

## Ethics Statement

This study was approved by the University of the Witwatersrand Human Research Ethics Committee (M150934 and M220673). All participants provided written informed consent.

## Conflicts of Interest

The authors declare no conflicts of interest.

## Supporting information


**Data S1:** Supporting Information.

## Data Availability

All statistical results are reported in this publication. R scripts and Stata do‐files used in the analyses are available from the corresponding author upon reasonable request.
