# Supplementary material for: Factors Associated With Risk Stratification and Overall Survival of Black South African Men With Non‐Metastatic Prostate Cancer
Source: Cancer Med. 2026 Feb 27;15(3):e71628. doi: 10.1002/cam4.71628 (PMC12949359; doi:10.1002/cam4.71628)
Supplement: Supplementary file 1 — Data S1: Supporting Information. [file CAM4-15-e71628-s001.docx]

**Supplementary Table 1: Multivariable Proportional Ordinal Regression on Prostate Cancer Non-Metastatic Risk Stratification (Low Risk =0, Intermediate Risk=1, High-Risk=2) and Associated Risk Factors.**

| Variable | Unadjusted OR (95% CI) | Unadjusted p-value | Adjusted OR (95% CI) | Adjusted p-value |
| --- | --- | --- | --- | --- |
| Age at diagnosis (in years) | 1.03 (1.01–1.05) | <0.001 | 1.04 (1.02–1.07) | <0.001 |
| **Minimal social support (single vs cohabiting)** | | | | |
| Single | 0.86 (0.63–1.17) | 0.334 | 1.16 (0.84–1.61) | 0.361 |
| **Employment status (unemployed vs employed)** | | | | |
| Unemployed | 1.31 (0.9–1.91) | 0.156 | 1.00 (0.65–1.52) | 0.985 |
| **Highest education attained (secondary or higher vs ≤ primary)** | | | | |
| Secondary or higher | 0.96 (0.72–1.28) | 0.765 | 0.98 (0.73–1.32) | 0.891 |
| **CVD and other comorbidity risk factors** | | | | |
| Diabetes (vs no diabetes) | 0.77 (0.53–1.12) | 0.173 | 0.83 (0.56–1.24) | 0.364 |
| Hypertension (vs no hypertension) | 0.92 (0.68–1.24) | 0.585 | 0.85 (0.61–1.18) | 0.324 |
| Living with HIV (vs without HIV) | 1.26 (0.81–1.97) | 0.314 | 1.50 (0.93–2.46) | 0.099 |
| Body mass index (per kg/m^2^) | 1.00 (0.97–1.02) | 0.850 | 1.01 (0.98–1.04) | 0.460 |
| Depression (psychological vs no distress) | 1.06 (0.72–1.59) | 0.763 | 0.99 (0.66–1.49) | 0.953 |
| **Behavioural risk factors** | | | | |
| **Alcohol consumption per week (heavy vs none to moderate)** | | | | |
| Heavy alcohol consumption | 1.01 (0.76–1.34) | 0.932 | 0.94 (0.69–1.27) | 0.682 |
| **Tobacco smoking (vs never)** | | | | |
| Currently yes | 0.96 (0.67–1.37) | 0.814 | 1.01 (0.68–1.49) | 0.968 |
| Yes (in the past) | 0.94 (0.67–1.31) | 0.707 | 0.89 (0.63–1.27) | 0.536 |

Data are presented as odds ratio (OR) and 95% confidence interval (CI). OR, 95% CI, and p values were obtained from proportional ordinal regression. Multivariable model adjusted for all listed variables. Reference categories are indicated in each domain.

**Supplementary Table 2: Multivariable Cox Proportional Hazards Regression on Overall Survival And Associated Risk Factors.**

| Variables | Deaths / Total participants | Unadjusted HR  (95% CI) | Unadjusted  p-value | Adjusted HR  (95% CI) | Adjusted  p-value |
| --- | --- | --- | --- | --- | --- |
| Age at diagnosis (in years) | 122/723 | 1.06 (1.03 - 1.08) | <0.001 | 1.05 (1.02–1.08) | <0.001 |
| **Minimal social support** | | | | | |
| Cohabitating | 92/515 | Reference |  | Reference |  |
| Single (including divorced and widowed) | 30/208 | 0.81 (0.54–1.23) | 0.322 | 0.75 (0.49–1.14) | 0.176 |
| Overall | 122/723 |  |  |  |  |
| **Employment status** | | | | | |
| Employed | 12/121 | Reference |  | Reference |  |
| Unemployed | 110/602 | 1.92 (1.06–3.48) | 0.032 | 1.24 (0.65–2.35) | 0.509 |
| **Highest education attained** | | | | | |
| Primary and below | 91/448 | Reference |  | Reference |  |
| Secondary or higher | 31/275 | 0.64 (0.42–0.96) | 0.030 | 0.72 (0.48–1.10) | 0.127 |
| Overall | 122/723 |  |  |  |  |
| **CVD and other comorbidity risk factors** | | | | | |
| Diabetes |  |  |  |  |  |
| Absent | 93/610 | Reference |  | Reference |  |
| Present | 29/113 | 1.77 (1.17–2.69) | 0.007 | 1.70 (1.08–2.67) | 0.022 |
| Overall | 122/723 |  |  |  |  |
| Hypertension |  |  |  |  |  |
| Absent | 32/225 | Reference |  | Reference |  |
| Present | 90/498 | 1.34 (0.90–2.01) | 0.152 | 1.08 (0.70–1.66) | 0.734 |
| Overall | 122/723 |  |  |  |  |
| Living with HIV |  |  |  |  |  |
| No | 109/636 | Reference |  | Reference |  |
| Yes | 13/87 | 0.98 (0.55–1.74) | 0.944 | 1.59 (0.84–3.01) | 0.155 |
| Overall | 122/723 |  |  |  |  |
| Body mass index (kgm^-2^) |  | 1.00 (0.96–1.03) | 0.824 | 1.00 (0.96–1.04) | 0.946 |
| Depression (psychological vs) |  |  |  |  |  |
| No distress | 93/620 | Reference |  | Reference |  |
| Psychological distress | 29/103 | 2.01 (1.33–3.05) | 0.001 | 1.67 (1.09–2.57) | 0.020 |
| Overall | 122/723 |  |  |  |  |
| **Behavioural risk factors** | | | | | |
| **Alcohol consumption per week** | | | | | |
| None to moderate | 58/371 | Reference |  | Reference |  |
| Heavy alcohol consumption | 64/352 | 1.21 (0.85–1.73) | 0.287 | 1.20 (0.83–1.75) | 0.330 |
| Overall | 122/723 |  |  |  |  |
| **Tobacco smoking (vs never)** | | | | | |
| Never | 32/242 | Reference |  | Reference |  |
| Currently yes | 32/210 | 1.12 (0.69–1.83) | 0.644 | 1.28 (0.76–2.16) | 0.360 |
| Yes (in the past) | 58/271 | 1.57 (1.02–2.42) | 0.040 | 1.37 (0.88–2.15) | 0.164 |
| Overall | 122/723 |  |  |  |  |
| **Nonmetastatic prostate cancer risk stratification** | | | | | |
| Low risk | 4/58 | Reference |  | Reference |  |
| Intermediate risk | 40/296 | 1.87 (0.67–5.23) | 0.232 | 1.65 (0.57–4.74) | 0.352 |
| High risk | 78/369 | 3.35 (1.23–9.16) | 0.018 | 2.51 (0.84–7.49) | 0.099 |
| Overall | 122/723 |  |  |  |  |
| Hormonal **treatment** | | | | | |
| Not received | 21/207 | Reference |  | Reference |  |
| Received | 101/516 | 1.99 (1.24–3.19) | 0.004 | 1.26 (0.72–2.22) | 0.417 |
| Overall | 122/723 |  |  |  |  |

Data are presented as hazards ratio (HR) and 95% confidence interval (CI). HR, 95% CI and p values obtained from Cox Proportional Hazards Regression on HR > 1 indicates higher hazard (i.e., worse overall survival). Reference categories are indicated per variable.

**Supplementary Table 3: Multivariable Cox Proportional Hazards Regression on Overall Survival and Associated Risk Factors Among 353 Participants aged 60 (57–63) years.**

| Variables | Unadjusted HR  (95% CI) | Unadjusted  p-value | Adjusted HR  (95% CI) | Adjusted  p-value |
| --- | --- | --- | --- | --- |
| Age at diagnosis (in years) | 1.05 (0.97–1.13) | 0.215 | 1.00 (0.92–1.08) | 0.967 |
| **Minimal social support (single vs cohabitating)** | | | | |
| Single (including divorced and widowed) | 0.76 (0.38–1.50) | 0.425 | 0.55 (0.26–1.17) | 0.119 |
| **Employment status (unemployed vs employed)** | | | | |
| Unemployed | 1.90 (0.88–4.09) | 0.103 | 1.83 (0.77–4.34) | 0.169 |
| **Highest education attained (secondary or higher vs ≤ primary)** | | | | |
| Secondary or higher | 0.90 (0.48–1.68) | 0.732 | 0.94 (0.49–1.81) | 0.852 |
| **CVD and other comorbidity risk factors** | | | | |
| Diabetes (vs no diabetes) | 3.39 (1.80–6.37) | <0.001 | 6.49 (2.83–14.86) | <0.001 |
| Hypertensive (vs no hypertension) | 1.50 (0.78–2.87) | 0.225 | 1.08 (0.51–2.28) | 0.841 |
| Living with HIV (vs without HIV) | 1.37 (0.65–2.88) | 0.401 | 1.75 (0.75–4.09) | 0.197 |
| Body mass index (kgm^-2^) | 0.95 (0.89–1.01) | 0.097 | 0.91 (0.85–0.98) | 0.015 |
| Depression (psychological vs no distress) | 1.94 (0.90–4.18) | 0.091 | 1.67 (1.09–2.57) | 0.020 |
| **Behavioural risk factors** | | | | |
| **Alcohol consumption per week (Heavy vs none to moderate)** | | | | |
| Heavy alcohol consumption | 0.83 (0.45–1.52) | 0.537 | 0.78 (0.40–1.55) | 0.483 |
| **Tobacco smoking (vs never)** | | | | |
| Currently yes | 2.48 (1.03–5.95) | 0.042 | 1.55 (0.60–4.02) | 0.370 |
| Yes (in the past) | 2.39 (1.00–5.73) | 0.051 | 1.92 (0.77–4.79) | 0.162 |
| **Nonmetastatic prostate cancer risk stratification** | | | | |
| Intermediate (vs low) | 3.84 (0.51–28.76) | 0.191 | 1.50 (0.18–12.82) | 0.711 |
| High (vs low) | 5.35 (0.72–39.59) | 0.100 | 1.04 (0.12–9.25) | 0.972 |
| **Treatment** | | | | |
| Hormonal treatment (yes vs no) | 4.77 (1.70–13.34) | 0.003 | 6.61 (2.01–21.70) | 0.002 |

Data are presented as odds ratio (OR) and 95% confidence interval (CI). OR, 95% CI, and p values were obtained from proportional ordinal regression. Multivariable model adjusted for all listed variables. Reference categories are indicated in each domain.

**Supplementary Table 4: Multivariable Cox Proportional Hazards Regression on Overall Survival and Associated Risk Factors Among 380 Participants aged 71 (68–75) years**

| Variables | Unadjusted HR  (95% CI) | Unadjusted  p-value | Adjusted HR  (95% CI) | Adjusted  p-value |
| --- | --- | --- | --- | --- |
| Age at diagnosis (in years) | 1.06 (0.99–1.14) | 0.115 | 1.06 (1.01–1.11) | 0.014 |
| **Minimal social support (single vs cohabitating)** | | | | |
| Single (including divorced and widowed) | 0.82 (0.42–1.60) | 0.561 | 0.78 (0.45–1.33) | 0.359 |
| **Employment status (unemployed vs employed)** | | | | |
| Unemployed | 1.91 (0.89–4.10) | 0.099 | 0.88 (0.31–2.46) | 0.800 |
| **Highest education attained (secondary or higher vs ≤ primary)** | | | | |
| Secondary or higher | 0.86 (0.46–1.60) | 0.625 | 0.56 (0.32–0.98) | 0.043 |
| **CVD and other comorbidity risk factors** | | | | |
| Diabetes (vs no diabetes) | 3.66 (1.97–6.79) | <0.001 | 1.10 (0.59–2.03) | 0.764 |
| Hypertensive (vs no hypertension) | 1.59 (0.83–3.05) | 0.160 | 0.95 (0.55–1.65) | 0.867 |
| Living with HIV (vs without HIV) | 1.28 (0.61–2.68) | 0.511 | 1.43 (0.49–4.21) | 0.515 |
| Body mass index (kgm^-2^) | 0.96 (0.90–1.01) | 0.135 | 1.02 (0.98–1.07) | 0.355 |
| Depression (psychological vs no distress) | 1.94 (0.90–4.18) | 0.091 | 1.84 (1.09–3.11) | 0.022 |
| **Behavioural risk factors** | | | | |
| **Alcohol consumption per week (Heavy vs none to moderate)** | | | | |
| Heavy alcohol consumption | 0.88 (0.48–1.60) | 0.666 | 1.28 (0.79–2.05) | 0.315 |
| **Tobacco smoking (vs never)** | | | | |
| Currently yes | 2.09 (0.91–4.81) | 0.083 | 1.01 (0.50–2.03) | 0.977 |
| Yes (in the past) | 2.00 (0.87–4.61) | 0.103 | 1.30 (0.77–2.19) | 0.320 |
| **Nonmetastatic prostate cancer risk stratification** | | | | |
| Intermediate (vs low) | 3.98 (0.53–29.77) | 0.178 | 1.38 (0.40–4.76) | 0.611 |
| High (vs low) | 5.23 (0.71–38.66) | 0.105 | 3.10 (0.86–11.24) | 0.084 |
| **Treatment** | | | | |
| Hormonal treatment (yes vs no) | 3.82 (1.51–9.70) | 0.005 | 0.68 (0.35–1.32) | 0.253 |

Data are presented as odds ratio (OR) and 95% confidence interval (CI). OR, 95% CI, and p values were obtained from proportional ordinal regression. Multivariable model adjusted for all listed variables. Reference categories are indicated in each domain.

**Supplementary Table 5: Stratified Multivariable Cox Proportional Hazards Regression Including Interactions Terms on Overall Survival And Associated Risk Factors Among All Participants**

| Variables | Unadjusted HR  (95% CI) | Unadjusted  p-value | Adjusted HR  (95% CI) | Adjusted  p-value |
| --- | --- | --- | --- | --- |
| Age at diagnosis (in years, centred) | 1.06 (1.03–1.08) | <0.001 | 1.02 (0.89–1.17) | 0.798 |
| **Minimal social support (single vs cohabitating)** | | | | |
| Single (including divorced and widowed) | 0.81 (0.54–1.23) | 0.322 | 0.77 (0.50–1.18) | 0.230 |
| **Employment status (unemployed vs employed)** | | | | |
| Unemployed | 1.92 (1.06–3.48) | 0.032 | 1.29 (0.68–2.44) | 0.438 |
| **Highest education attained (secondary or higher vs ≤ primary)** | | | | |
| Secondary or higher  *Main effect*  *Interaction effect with age (centred)* | 0.64 (0.42–0.96)  1.00 (0.94–1.06) | 0.030  0.999 | 0.74 (0.48–1.13)  1.00 (0.94–1.05) | 0.164  0.860 |
| **CVD and other comorbidity risk factors** | | | | |
| Diabetes (vs no diabetes)  *Main effect*  *Interaction effect with age (centred)* | 1.77 (1.17–2.69)  1.01 (0.94–1.08) | 0.007  0.882 | 2.09 (1.30–3.36)  0.91 (0.85–0.97) | 0.002  0.004 |
| Hypertensive (vs no hypertension) | 1.34 (0.90–2.01) | 0.152 | 1.00 (0.65–1.55) | 0.983 |
| Living with HIV (vs without HIV) | 0.98 (0.55–1.74) | 0.944 | 1.54 (0.80–2.96) | 0.192 |
| Body mass index (kgm^-2^)  *Main effect*  *Interaction effect with age (centred)* | 1.00 (0.96–1.03)  1.01 (1.00–1.01) | 0.824  0.001 | 0.99 (0.95–1.03)  1.00 (0.99–1.01) | 0.622  0.095 |
| Depression (psychological vs no distress) | 2.01 (1.33–3.05) | 0.001 | 1.76 (1.15–2.70) | 0.010 |
| **Behavioural risk factors** | | | | |
| **Alcohol consumption per week (Heavy vs none to moderate)** | | | | |
| Heavy alcohol consumption | 1.21 (0.85–1.73) | 0.287 | 1.10 (0.75–1.62) | 0.617 |
| **Tobacco smoking (vs never)** | | | | |
| Currently yes | 1.12 (0.69–1.83) | 0.644 | 1.21 (0.71–2.05) | 0.493 |
| Yes (in the past) | 1.57 (1.02–2.42) | 0.040 | 1.40 (0.90–2.20) | 0.139 |
| **Nonmetastatic prostate cancer risk stratification** | | | | |
| Intermediate (vs low) | 1.87 (0.67–5.23) | 0.232 | 1.35 (0.46–3.94) | 0.581 |
| High (vs low) | 3.35 (1.23–9.16) | 0.018 | 2.02 (0.67–6.09) | 0.210 |
| **Treatment** | | | | |
| Hormonal treatment (yes vs no)  *Main effect*  *Interaction effect with age (centred)* | 3.82 (1.51–9.70)  1.04 (1.01–1.08) | 0.005  0.004 | 1.72 (0.90–3.30)  0.92 (0.87–0.98) | 0.101  0.009 |

Data are presented as odds ratio (OR) and 95% confidence interval (CI). OR, 95% CI, and p values were obtained from proportional ordinal regression. Multivariable model adjusted for all listed variables. Reference categories are indicated in each domain.


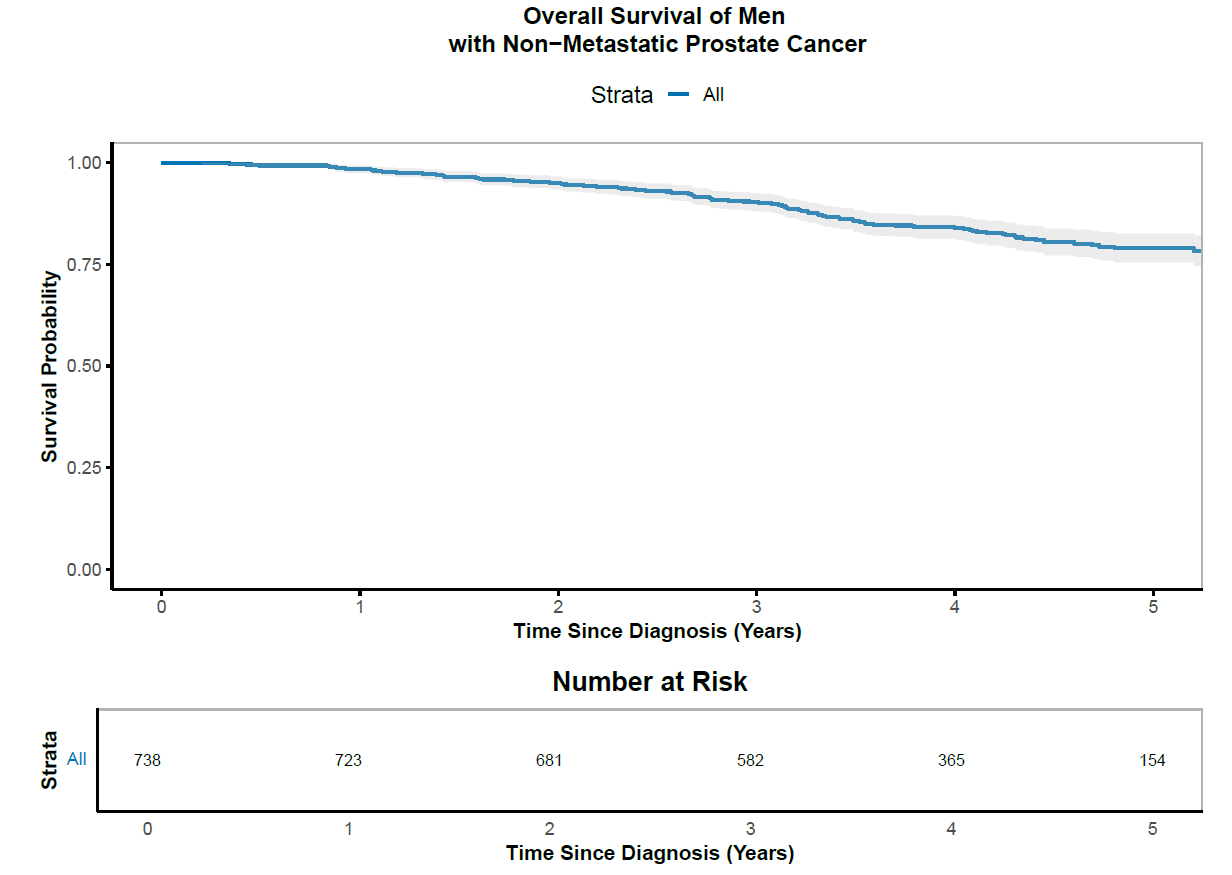


**Supplementary Figure 1: Kaplan Meir Curve on overall survival**. Number at risk for the 5-year follow up are shown in the Strata.
